# Neighborhood Disadvantage Is Associated with Depressive Symptoms but Not Depression Diagnosis in Older Adults

**DOI:** 10.3390/ijerph17165745

**Published:** 2020-08-08

**Authors:** Courtney J. Bolstad, Reagan Moak, Cynthia J. Brown, Richard E. Kennedy, David R. Buys

**Affiliations:** 1 Department of Psychology, Mississippi State University, Mississippi State, MS 39762, USA; 2 Department of Food Science, Nutrition, and Health Promotion, Mississippi State University, Mississippi State, MS 39762, USA; rm1644@msstate.edu (R.M.); david.buys@msstate.edu (D.R.B.); 3 Division of Gerontology, Geriatrics, and Palliative Care and Integrative Center for Healthy Aging, University of Alabama Birmingham, Birmingham, AL 35294, USA; cynthiabrown@uabmc.edu (C.J.B.); rekenned@uab.edu (R.E.K.); 4 Birmingham/Atlanta VA Geriatric Research, Education, and Clinical Center, Birmingham, AL 35233, USA

**Keywords:** neighborhood disadvantage, depression diagnosis, depressive symptomology, healthcare access, health disparities

## Abstract

Disadvantaged neighborhood environments may have low access to healthcare, perpetuating health disparities. Previous research has reported on associations between neighborhood disadvantage (ND) and depressive symptomology but not depression diagnoses, which may indicate access to healthcare. This study tested how ND relates to depressive symptomology and diagnosis to assess for neighborhood disparities in mental health care cross-sectionally. Data from 998 community-dwelling, Black and White individuals aged 65+ included in the University of Alabama at Birmingham Study of Aging were analyzed. We obtained participants’ depressive symptomology from the Geriatric Depression Scale (*n* = 100) and a verified depression diagnosis from self-report and review of medication, physician-report, and/or hospital discharge summaries (*n* = 84). We assessed ND from US Census data, divided the sample into tertiles of ND and fit models with Generalized Estimating Equations covarying for various other variables (e.g., sex, race, physical performance, socioeconomic status, etc.). We found living in the high and mid-ND tertiles to be associated with depressive symptomology, yet ND had no significant relation to depression diagnosis. Therefore, older adults living in high and mid-disadvantaged neighborhoods may be more likely to experience depressive symptomology but not receive a diagnosis, indicating a possible disparity in mental health care.

## 1. Introduction

Where people live matters for their health. Research in sociology and public health have long demonstrated that resources and risks within neighborhoods and communities can impact an individual’s health status, and also that low socioeconomic status among people in a neighborhood is associated with higher levels of risk or hazard for individuals in that neighborhood, independent of a person’s own disadvantage [1,2,3]. This suggests neighborhood factors contribute to health over and above the characteristics inherent to the individuals living there.

While these findings have been shown in the general population, the focus of the present study is on older adults, a population which may be more susceptible to their environment than other populations [4]. The neighborhood can be either a social resource, a source of risk for adverse outcomes, or some combination of both [5], especially for special populations such as older adults. 

These deleterious environments may affect mental health outcomes. Living in highly disadvantaged neighborhoods has also been associated with depressive symptomology among adults [6]. Furthermore, the neighborhood environments where older adults live are increasingly being recognized as an important factor in the aging process for psychological outcomes [7]. Given the increased risk of mental health challenges for individuals living in disadvantaged neighborhoods, one would expect greater healthcare resources to be allocated to those environments. However, Kirby and Kaneda [8] found neighborhood disadvantage (ND) was associated with a lack of access to healthcare. 

While previous research has reported on associations between ND and depressive symptoms [6] and on neighborhood characteristics and access to healthcare among adults [8], no work to date has examined if neighborhood characteristics are associated with a verified diagnosis of depression in adults of any age, which may be a marker of access to mental health care. Given this gap in research, the purpose of this paper is to explore, among the older adult population, if ND is associated similarly with both depressive symptoms and verified depression diagnosis. If there are gaps in the point estimates generated by these models, we will have further evidence that ND may be associated with disparities in both mental health and access to mental health care.

## 2. Materials and Methods

### 2.1. Sources of Data

For this cross-sectional analysis, we used data from the University of Alabama at Birmingham (UAB) Study of Aging (SOA), an observational, longitudinal study of 1000 Medicare beneficiaries. Due to the exploratory nature of this secondary analysis, a power analysis was not used to determine the sample size. At baseline (between 1999–2001), participants were community-dwelling persons aged 65 and older living in five rural and urban counties in central Alabama. The simple random sample within strata was balanced at baseline in order to reduce sampling bias for Blacks (50% Black vs. White), males (50% male vs. female), and rural residents (51% rural vs. urban). Standardized questionnaires designed to assess mobility, social and demographic information, medical history, and health service utilization were collected at baseline in the home and in subsequent telephone follow-up interviews conducted every six months. At baseline, a survey was sent to participants’ physicians for verification of medical conditions, and records from hospitalizations for three previous years were also obtained [9,10]. 

Additionally, participants’ addresses were geo-coded, and the census tract characteristics were linked to data from the 2000 United States Census Summary Files 1 and 3. Characteristics available in these files at the census tract level were used to compute the neighborhood disadvantage index (NDI) as described by Ross, Mirowsky, and Pribesh [11]. While using census tracts as a measure of neighborhood is limited by the fact that residents are not likely to recognize the seemingly invisible boundaries that are used for their demarcation [1], this level of measurement has been identified as an adequate and accepted tool for assessing neighborhood characteristics due to the limited availability of other data on neighborhoods [12]. The census tract has also been shown to detect socioeconomic gradients and neighborhood differences in the general population including populations of older adults [13]. Before the project was initiated, the UAB Institutional Review Board reviewed and approved the UAB SOA. 

### 2.2. Independent Variable

#### Neighborhood Disadvantage

Neighborhood disadvantage was measured at the census tract level where participants lived. This variable was conceptualized using the NDI [11], which includes prevalence rates of poverty and female-headed households in the census tract. Prevalence of poverty is a marker of economic disadvantage and prevalence of single-mother households is a marker of social disadvantage. In keeping with Ross, Mirowsky and Pribesh [11], the rates of poverty and female-headed households were divided by 10 and the mean of these values was assigned to individuals as the NDI. A one-unit increase in the NDI represents a 10 percent increase in the prevalence of poverty and female-headed households at an individual’s census tract level. Persons in this study lived among 166 census tracts with 45 of the tracts having only one person represented in this study, 73 tracts had two, three, or four persons in the study living in the same tract, and 48 tracts had five or more persons in the tract. In this sample, the mean census tract rate of poverty was 17.2% with a minimum of 0.3% and a maximum of 58.6%. The mean rate of female-headed households was 9.7%, with a range of 1.4% to 33.5%. In order to examine how depressive symptoms and verified depression diagnoses differed based on ND, participants were divided by level of ND into three nearly equal groups (tertiles), broken at the point nearest one-third of the sample with a different NDI value. Groups were labeled as low disadvantaged (*n* = 337), mid disadvantaged (*n* = 331), and high disadvantaged (*n* = 330). Dividing the independent variable (ND levels in our analysis) into tertiles is commonly used in exploratory data analysis to examine the relation between predictors and outcome measures when the nature of the relationship (e.g., linear versus nonlinear) is not known [14]. 

### 2.3. Dependent Variables

#### 2.3.1. Depressive Symptomology 

The Geriatric Depression Scale short form (GDS) is a 15-item questionnaire used to capture depressive symptoms [15], with scores ranging from 0 to 15. Affirmative responses on more than 5 questions is suggestive of the participant experiencing depression. For analytical purposes, this was coded as a dichotomous variable with persons scoring greater than 5 being assigned a value of 1 for elevated depressive symptoms (*n* = 100, 10%) and all others being assigned a value of 0. The GDS short form has been found to be reliable (α = 0.75) and valid in previous research [15,16], and the internal consistency of the GDS short form in the present study was adequate (α = 0.73). 

#### 2.3.2. Verified Depression Diagnosis 

Depression diagnosis was verified with any of the following techniques: (1) Self-reported physician diagnosis and medication (*n* = 38, 4%), (2) Physician-reported diagnosis on a survey about the participant (*n* = 52, 5%), or (3) Documented diagnosis on a hospital discharge summary within three years (*n* = 10, 1%). Sixteen persons had verification in more than one category, and 84 persons had verification according to at least one of these criteria. 

### 2.4. Covariates

Several covariates were included in our analyses. Perceived neighborhood disorder was measured using participants’ responses to a single question: “Do you limit your activities for fear of being robbed or attacked?” Poverty, an important variable used to distinguish the effects of individual disadvantage from ND, was conceptualized using the 2000 Federal Poverty Guidelines as a framework for setting cut points. Persons living alone with an income of USD 8350 or persons living with someone with an income of less than USD 11,250 were considered poor according to that measure; therefore, we assigned poverty status to individuals living alone with an income of less than USD 8000 and persons living with someone making less than USD 12,000 due to the categories available in the UAB SOA. Additionally, age was reported in years at baseline and was used as a continuous variable. Sex was included (1 = female), and race was reported as White (coded 1) and Black (coded 0). The UAB SOA was designed specifically to assess differences between older Black and White adults, and therefore only older Black and White adults were sampled. The UAB SOA sample was not intended to be representative of any specific geographic region. We also adjusted for participants having an education of less than 7 years and those who lived alone. 

Physical functioning was measured using the Short Physical Performance Battery (SPPB) which includes timed tests of standing balance, walking, and the ability to rise from a chair [17]. Each of these tests were scored from 0 to 4, with 0 representing inability to perform the task and 4 indicating best performance. Composite scores for this measure were calculated as the sum of each individual task and ranged from 0 to 12, with higher scores indicating better physical performance. The SPPB has been found to have adequate reliability (α = 0.76) and validity in previous research with older adults [17,18], and the internal consistency of the SPPB in the present study was adequate (α = 0.78).

### 2.5. Analytic Sample and Technique

The final analytic sample was comprised of 998 participants as one participant was missing data on depressive symptomology and another on poverty, which resulted in exclusion from the analyses. Descriptive statistics were calculated for all variables by ND strata. Differences in the variables between strata were assessed using ANOVA or chi-square tests, and full models were fit using generalized estimating equations [19] which account for the nesting of cases in a second-level variable, which in this case is neighborhoods measured by census tracts. 

## 3. Results

Descriptive statistics are depicted in Table 1, both by ND strata and the total sample. Depressive symptomology was more prevalent in mid and high disadvantaged neighborhoods, although there was no difference between the ND strata regarding verified depression diagnosis. Participants who lived in mid and high disadvantaged neighborhoods tended to live in more rural areas, and more Black participants resided in these neighborhoods. Further, mid and high disadvantaged neighborhoods also had higher rates of participants endorsing living below poverty, limiting their activities due to fear of being robbed or attacked, and having less than a 7th grade education. Participants in the low disadvantaged neighborhoods had significantly better physical performance. There were no differences between the rates of living alone or being female among the ND strata. Regarding the concordance between elevated depressive symptoms and verified depression diagnosis, 28 participants with depressive symptoms also had a verified depression diagnosis, 72 participants with depressive symptoms did not have a verified depression diagnosis, and 56 participants with a verified depression diagnosis did not report elevated depressive symptoms. 

Table 2 shows the results of the generalized estimating equations. In the adjusted models, participants in mid and high disadvantaged neighborhoods had approximately 1.7 greater odds (2.3 unadjusted) of endorsing significant depressive symptoms than participants in low disadvantaged neighborhoods; however, ND was not significantly related to having a verified depression diagnosis in adjusted or unadjusted models. When analyzing how our outcome measures relate to the covariates in the adjusted model, we found poverty, race, and physical performance to be significantly related to both depressive symptoms and diagnosis. Specifically, participants who lived below poverty had 2.5 greater odds (3.1 unadjusted) of reporting depressive symptoms and 1.6 greater odds (1.2 unadjusted, *p* > 0.05) of having a verified depression diagnosis compared to participants who did not live below poverty. Additionally, Black participants had 61% lower odds (1.4 greater odds when unadjusted, *p* > 0.05) of reporting depressive symptoms and 81% lower odds (60% unadjusted) of having a verified depression diagnosis than White participants. Participants with better physical performance had 20% lower odds (21% unadjusted) of reporting depressive symptoms and 14% lower odds (10% unadjusted) of having a depression diagnosis than participants with worse physical health. Further, female participants had 1.7 greater odds (1.9 unadjusted) of having a depression diagnosis than male participants, but this difference was not found for depressive symptomology. Finally, we found participants who endorsed limiting their activities due to fear of being robbed or attacked, which is a proxy for perceived neighborhood disorder, had 2.7 greater odds (2.9 unadjusted) of reporting significant depressive symptoms than participants who did not limit their activities, but there were no significant differences between these groups when considering verified depression diagnosis.

In order to explore the potential interaction between ND and race on depressive symptoms and verified depression diagnoses, post-hoc interaction analyses were conducted. The interaction terms in both models were not significant (all *p*s > 0.082), indicating that endorsement of significant depressive symptoms or having a depression diagnosis was not differentially affected by race at the three levels of ND.

## 4. Discussion

The present study explored if ND was associated similarly with both depressive symptoms and diagnosis among older adults. We found that among older adults in adjusted analyses, living in a mid or high disadvantaged neighborhood (relative to low disadvantage) was associated with more than a 70% greater likelihood of having depressive symptoms but was not associated with having a verified depression diagnosis. These outcomes may represent disparities between people who live in disadvantaged contexts compared to those who do not. These disparities may include access to mental health care and/or cultural bias against reporting depressive symptoms to healthcare providers. Findings from this study may suggest individuals in disadvantaged neighborhoods may accept their depressive symptoms as routine and not see the need for professional intervention, although this was not directly tested in the present study. On the other hand, individuals in disadvantaged neighborhoods may recognize their depressive symptoms but do not have access to providers that would diagnose and treat the conditions. Further, it is possible providers see depressive symptoms in individuals from disadvantaged neighborhoods as justifiable and therefore do not diagnose or treat the symptoms.

Our findings also show persons living below poverty were more likely to report having both depressive symptoms and a diagnosis. This may indicate that even though persons in poverty are getting a diagnosis, they are early in their treatment, not getting treated, or their treatment is not effective. Perhaps living below poverty is a perpetuating cause of depression, and treatment may not address the factors associated with living below poverty [20]. In addition to those living in poverty, individuals in disadvantaged neighborhoods were also more likely to experience depressive symptoms. However, living in poverty was associated with a verified depression diagnosis, while ND was not. We believe this may be due to the individual versus community level nature of how these variables were operationalized in the present study, respectively. Specifically, poverty was determined by an individual’s annual income, whereas ND was defined by census tract. Therefore, people living in disadvantaged neighborhoods are not necessarily also living below poverty, although it is possible. The difference in diagnosis, but not symptoms, may be due to people living in poverty underreporting depressive symptoms to providers, which has been reported elsewhere [21]. Underreporting may occur for various reasons, including being unable to afford treatment if diagnosed. However, this remains an empirical question as this was not tested in the present study. Blacks were less likely to have both depressive symptoms and diagnoses. It is possible that differences in religiosity and familial contact between Blacks and Whites may mediate the relation between race and depressive symptoms and diagnosis [22,23]. Additionally, analyses on the interaction between ND and race on depressive symptoms and verified depression diagnosis were not significant. Previous research has found that racial disparities often narrow or dissipate when social contexts, such as ND, are equivalent between the racial groups [24,25]. Further, better physical performance was associated with both a lower likelihood of depressive symptoms and diagnosis. This may be the result of individuals with better physical functioning being able to engage in more positive reinforcement activities (e.g., visiting friends and family, doing outdoor recreational activities, etc.), which are known to combat depressive symptoms [26]. 

In the same adjusted models, females were more likely to have a verified depression diagnosis than men but were not more likely to report depressive symptoms. This finding is most likely the result of older men being resistant to seek treatment and older women being more proactive in seeking care [27]. Additionally, our results show persons who reported limiting their activities for fear of being robbed or attacked, which is a proxy measure for perceived neighborhood disorder, were more likely to report depressive symptoms but were not more likely to have a diagnosis. People who live in disordered neighborhoods may experience fear and anxiety when participating, or considering participating, in activities. This may lead individuals to reduce their activities as a direct result of the anxiety involved, and/or individuals may find activities less pleasurable due to the fear or anxiety, which may lead to depressive symptoms [26,28]. The finding regarding no greater likelihood of receiving a diagnosis, on the other hand, may represent a disparity in access to mental health care or a cultural bias against reporting depressive symptoms to healthcare providers among individuals in disordered neighborhoods. 

## 5. Strengths and Limitations

One of the several strengths of this study was the examination of the correlation between ND and depression-related results in a heterogeneous population of community-dwelling older adults located in 180 unparalleled neighborhoods. This study provided detailed data concerning the socioeconomic status, social support, and psychological factors at the level of the individual in addition to ND at the level of the community. Validity was ensured through the integrity of the outcomes and the robust data utilized for these conclusions. 

Along with great strengths, our study also contained limitations. The cross-sectional data used were unable to account for changes occurring over time or the directionality of cause, which is a common problem found in the literature on the relation between health and neighborhood effects [29]. This means we were unable to assess the development of depression due to neighborhood characteristics or to rule out reverse causation (i.e., depressive symptoms, health, or demographic characteristics may limit one from moving out of a disadvantaged neighborhood [29,30]). Additionally, we were unable to account for the possibility that the GDS assessed the recent onset of depressive symptoms before participants had the opportunity to report these symptoms to their physicians in order to receive a verified diagnosis. The data also focused on the age group of older adults (i.e., 65 years of age and older), which means we were unable to stratify the effects and progression of depression among younger age groups. Further, restricting our analyses to older adults excluded younger life transitions that could lead to depression. We were also unable to generalize our findings to other geographical regions since this study was performed in the southern United States. Additionally, it was inappropriate to hierarchically model neighborhoods due to nesting of few participants in some neighborhoods. This limitation is described elsewhere [31]; this would not allow us to separate out the variation of depression outcomes inferential to the neighborhood level. Finally, the present study did not assess several key moderating variables between experiencing depressive symptoms and obtaining a verified diagnosis, which limits our ability to understand why diagnoses of depression were not given to individuals with symptomology. These moderating variables include individuals’ frequency of reporting symptoms and physicians’ assessment of depressive symptoms. Future research examining these variables in respect to individuals’ ND will further elucidate the possible moderators of mental health disparities experienced by individuals in disadvantaged neighborhoods.

## 6. Future Directions

In the future, we hope to examine the effects of ND on residents with depression-related health outcomes in the UAB SOA, as well as larger databases, to allow for hierarchical models that contain assessments over time to further determine the correlation and potential causation between ND and depressive related results. We would also like to include a range of age groups to stratify samples by age to determine if ND primarily or asymmetrically affects older adults. Future policy implications will be considered to encourage alteration in dynamics of communities for the betterment of their residents. In the future, programs and services should be tailored to an individual’s resources and specific neighborhood. For example, if an individual presents depressive symptoms, the cause may be traced back to their neighborhood if they are known to live in a disadvantaged area. By providing health services or lifestyle alterations, depression can be controlled by an individual’s living conditions which may improve treatment efficiency and effectiveness as well as remission. Lastly, further work should be completed to determine how to effectively intervene in one’s community as well as with an individual. For instance, to improve resources for those who suffer from depression, a professional may be provided to reduce any causes from the neighborhood to improve daily conditions for the patient. In addition, using ND to help in the diagnosis of depression could locate a large factor as well as determine the most effective method of treatment on a patient-by-patient basis to treat the person rather than the mental illness. We hope to broaden our variables of ND to find a more parsimonious model to examine these cases of depression that are undetected by investigating more sociodemographic characteristics and available health services in order to increase accurate diagnosis, treatment, and remission of depression with the overall goal of reducing the prevalence of depression. 

## 7. Conclusions

This study demonstrates that the degree of disadvantage in a neighborhood, as well as other individual and neighborhood characteristics, are associated with depressive symptomology but not depression diagnoses in older adults. This provides further evidence of healthcare disparities and a rationale for increased mental health services in disadvantaged neighborhoods, especially for older adults.

## Figures and Tables

**Table 1:** Participant and neighborhood characteristics by level of neighborhood disadvantage.

Variables	Low Disadvantage	Mid Disadvantage	High Disadvantage	Total
	*n* = 337	*n* = 331	*n* = 330	*n* = 998
Independent Variables				
Persons Below Poverty ***	57 (17%)	145 (44%)	141 (43%)	343 (34%)
Rural ***	106 (32%)	216 (65%)	190 (58%)	512 (51%)
Limit activities for fear of being robbed/attacked ***	37 (11%)	67 (21%)	72 (22%)	176 (18%)
Black ***	80 (24%)	182 (55%)	236 (72%)	498 (50%)
Female	163 (49%)	171 (52%)	164 (50%)	498 (50%)
Live alone	93 (28%)	114 (35%)	110 (33%)	317 (32%)
Education < 7th Grade ***	34 (10%)	96 (29%)	74 (22%)	204 (20%)
Physical Performance ***	7.6 (3.3)	6.5 (3.2)	6.5 (3.2)	6.9 (3.2)
Dependent Variables				
Depressive Symptomology (GDS > 5/15) **	21 (6%)	43 (13%)	36 (11%)	100 (10%)
Verified Depression Diagnosis	29 (9%)	27 (8%)	28 (9%)	84 (8%)

Note. ** *p* ≤ 0.01, *** *p* ≤ 0.001; Persons Below Poverty refers to participants below poverty line 200% of the federal poverty guideline. Physical Performance refers to the Short Physical Performance Battery *M* (*SD*). GDS: Geriatric Depression Scale short form.

**Table 2:** Generalized Estimating Equation Results for Neighborhood Disadvantage Effects on Depression-related Outcomes (*n* = 998).

Variable	Depressive Symptomology			Depression Verified		
	Unadjusted OR	Adjusted OR (CI)		Unadjusted OR	Adjusted OR (CI)	
Mid-Disadvantage	2.32 ***	1.75 *	(1.04, 2.94)	0.99	1.42	(0.79, 2.56)
High-Disadvantage	2.01 **	1.71 *	(1.09, 2.68)	0.96	1.85	(0.98, 3.47)
Lives Below Poverty	3.13 ***	2.47 ***	(1.61, 3.78)	1.15	1.60 *	(1.00, 2.58)
Rural	1.49 *	0.97	(0.67, 1.41)	0.81	0.60	(0.36, 1.01)
Limit Activities	2.92 ***	2.68 ***	(1.74, 4.12)	1.21	1.46	(0.88, 2.43)
Black	1.38	0.39 ***	(0.23, 0.65)	0.40 ***	0.19 ***	(0.11, 0.32)
Female	1.11	0.78	(0.50, 1.20)	1.92 **	1.74 *	(1.05, 2.88)
Lives Alone	1.24	0.99	(0.56, 1.74)	0.90	0.70	(0.44, 1.11)
Education < 7th Grade	1.61	1.27	(0.62, 2.57)	0.45 *	0.71	(0.35, 1.44)
Physical Performance	0.79 ***	0.80 ***	(0.74, 0.87)	0.90 ***	0.86 ***	(0.80, 0.92)

Note. * *p* ≤ 0.05, ** *p* ≤ 0.01, *** *p* ≤ 0.001; OR: odds ratio; CI: confidence interval; Limit Activities refers to participants endorsement of limiting their activities due to fear of being robbed or attacked.
